# Electronic Nose for Monitoring Odor Changes of *Lactobacillus* Species during Milk Fermentation and Rapid Selection of Probiotic Candidates

**DOI:** 10.3390/foods9111539

**Published:** 2020-10-26

**Authors:** Zoltan Kovacs, Zsanett Bodor, John-Lewis Zinia Zaukuu, Timea Kaszab, George Bazar, Tamás Tóth, Csilla Mohácsi-Farkas

**Affiliations:** 1 Department of Measurement and Process Control, Faculty of Food Science, Szent István University, Somlói út 14–16, H-1118 Budapest, Hungary; bodor.zsanett@hallgato.uni-szie.hu (Z.B.); zaukuu.john-lewis.zinia@hallgato.uni-szie.hu (J.-L.Z.Z.); kaszab.timea@szie.hu (T.K.); 2 Department of Nutritional Science and Production Technology, Faculty of Agricultural and Environmental Sciences, Szent István University, Guba S. út 40, H-7400 Kaposvár, Hungary; bazar@agrilab.hu; 3 ADEXGO Kft., Lapostelki út 13, H-8230 Balatonfüred, Hungary; tamas.toth@adexgo.hu; 4 Department of Microbiology and Biotechnology, Faculty of Food Science, Szent István University, Somlói út 14–16, H-1118 Budapest, Hungary; mohacsine.farkas.csilla@szie.hu

**Keywords:** yogurt, gram-positive, biofortification, artificial aroma sensing, chemometric

## Abstract

Probiotic bacteria have been associated with a unique production of aroma compounds in fermented foods but rapid methods for discriminating between foods containing probiotic, moderately probiotic, or non-probiotic bacteria remain aloof. An electronic nose (e-nose) is a high-sensitivity instrument capable of non-invasive volatile measurements of foods. In our study, we applied the e-nose to differentiate probiotic, moderately probiotic, and non-probiotic *Lactobacillus* bacteria strains at different fermentation time points (0th, 4th, and 11th) of milk fermentation. The pH of the changing milk medium was monitored with their corresponding increase in microbial cell counts. An e-nose with two gas chromatographic columns was used to develop classification models for the different bacteria groups and time points and to monitor the formation of the aromatic compounds during the fermentation process. Results of the e-nose showed good classification accuracy of the different bacteria groups at the 0th (74.44% for column 1 and 82.78% for column 2), the 4th (89.44% for column 1 and 92.22% for column 2), and the 11th (81.67% for column 1 and 81.67% for column 2) hour of fermentation. The loading vectors of the classification models showed the importance of some specific aroma compounds formed during the fermentation. Results show that aroma monitoring of the fermentation process with the e-nose is a promising and reliable analytical method for the rapid classification of bacteria strains according to their probiotic activity and for the monitoring of aroma changes during the fermentation process.

## 1. Introduction

Milk is an important dairy product from mammalian sources such as cows, goats, buffalo, or sheep. Its fermented form has yielded many different foods in the dairy industry such as ice cream, cheese, yogurt, etc. Fermentation of milk into food products is often associated with starter cultures such as *Streptococcus thermophilus* and *Lactobacillus delbrueckii* subsp. *bulgaricus* [1]. *Lactobacilli* are gram-positive bacteria consisting of both anaerobic and aerobic species. The main product of their fermentation is lactic acid but secondary products are also generated as diacetyl, acetoin, and acetone [2]. *Lactobacillus* strains have significant industrial importance due to their fermentation activity and health benefits such as maintaining the good condition of the gastrointestinal tract (GIT). Nowadays, a healthy lifestyle including special diets has an important role in consumers’ choices [3]. Therefore, due to the increasing demand for yogurt and dairy products, the necessity to isolate new *Lactobacillus* strains has increased. Probiotics are microorganisms that can survive in the stomach and upper GIT and express their health benefits in the lower GIT [2]. Their major benefits include supporting the immune system, competing with pathogenic microorganisms, and serving as a nutrient growth factor [4].

The selection of a starter culture is an important issue in the dairy industry as it determines the probiotic activity, taste, aroma, and texture, etc. of the product. The main determining factor for the flavor and scent of fermented dairy products is the type of the applied bacteria strain. Different bacteria strains, working with others, have different rates of producing important metabolites like lactic acid and acetaldehyde. Acetaldehyde is one of the key aromas in fermented milk but more than 90 flavor compounds have also been detected in milk products [5,6]. Therefore, differentiating between probiotic and non-probiotic strains is crucial for the production of fermented products and this can easily be done by analyzing the differences in their aroma formation during fermentation. 

Many techniques have been explored in this regard involving small-angle neutron scattering [7], enzymatic hydrolysis, and assessment of physical parameters [8]. One of the most common approaches for the determination of probiotic activity is to look for specific genes or test the survival ability and resistance to a low pH environment [4]. These methods are expensive and require a longer duration and greater knowledge. Therefore, there is a need to develop new methods that provide a rapid and cheaper differentiation of the different bacteria strains [9].

Sensory analysis of products can be a useful tool in choosing the appropriate strain or even could be used for differentiating between strains with different probiotic activity. Instrumental sensory analysis such as the application of an electronic nose (e-nose) could serve as an objective and rapid tool for describing the changes in aroma production during fermentation of food products. An electronic nose is an analytical instrument equipped with artificial sensors for detecting volatile compounds in food with higher sensitivity and accuracy compared to the human nose. The instrument has been used for monitoring fermentation in different food products such as tea [10], meat and fish [11], and yogurt [12], and with other tools for the evaluation of the effect of feed supplementation on sensory properties of raw milk [13]. Swiss researchers used mass spectrometry (MS)-based e-nose to differentiate between different *Lactobacillus casei* species. Their study showed that the MS-based e-nose was able to differentiate between relatively similar strains and predict the aroma of the products [14]. Swedish researchers also successfully used the e-nose and near-infrared spectroscopy for monitoring the fermentation of yogurt [15,16]. They reported that the response of the e-nose sensor was proportional to galactose production or inversely proportional to the pH and the onset of coagulation could be observed. Their study did not primarily focus on identifying volatile compounds but to present a new perspective for quality control of fermented milk products. 

The objective of this study was to develop classification models based on the results of the electronic nose for the differentiation of *Lactobacillus bulgaricus* bacteria strains according to their probiotic activity at different time points of milk fermentation. In addition, models were built with the electronic nose to rapidly predict and monitor some major microbial characteristics of the bacteria strains and the main aroma components produced during the fermentation.

## 2. Materials and Methods

### 2.1. Analyzed Bacteria Strains

Fifteen different types of *Lactobacillus* bacteria strains were acquired in a freeze-dried state from reputable sources and separately used to produce fermented milk products based on their probiotic grouping. Depending on the growth rate, optical density, biomass production, minimal inhibitory concentration of bile, and best recovery after 3 h at low pH and pepsin, the bacteria strains could be grouped into non-probiotic (S2, S3, S4, S29, S30), moderately probiotic (S1, S7, S8, S9, Y12), and probiotic (R1, S6, S10, S11, S22) [9]. All the species used in this study were *Lactobacilli bulgaricus*. They ferment the glucose to lactic acid as a major end product by homolactic fermentation (Embden-Meyerhof-Parnas pathway and subsequent pyruvate reduction). 

### 2.2. Preparation of the Fermented Milk Product

#### 2.2.1. Preparation of Reconstituted Milk

Skimmed milk powder for microbiological use was acquired from Sigma Aldrich (St. Louis, Missouri, USA) and used in this study. According to the producer, the skimmed milk powder contained 4.7–6.0% total nitrogen (N), ≤1.5% lipid, ≥50.0% reducing sugars (as lactose monohydrate), and ≤ 5% loss on drying. On each experimental day, the skimmed milk powder was freshly diluted to 10% *w*/*v* in sterilized distilled water until it reached 3.5% protein content as recommended by the producer. For purposes of this study, the resulting mixture was referred to as reconstituted milk and could be said to contain <1% ash, <0.15% lipid, 0.47–0.6% total nitrogen, and about 5% reducing sugar content per the calculations after the 10% *w*/*v* dilution. 

#### 2.2.2. Preparation of Strain Suspension (Activation of Freeze-Dried Bacteria)

The freeze-dried bacteria strain (10 mg) was weighed into a 10 mL flask then filled up to volume with the reconstituted skimmed milk. This was referred to as the strain suspension and was cultured for 24 h at 37 °C to obtain the activated bacteria before initial cell number counting was performed. Cell numbers were determined using the Breed staining method [17] with a light microscope at 100-times magnitude with immersion oil in three parallel repeats. Each of the 15 *Lactobacillus* bacteria strains suspension was prepared on different days using a random design.

#### 2.2.3. Preparation of the Milk Suspension

The activated strain suspension was further diluted with reconstituted milk to set the initial cell count of the milk suspension at 10^6^ CFU/mL using the average cell number determined by the Breed staining method. This sample preparation was applied to provide similar conditions for the milk fermentation for all 15 bacteria strains having different growing characteristics. A total of 400 mL of the milk suspension was prepared for the entire study for each of the 15 strains on different days.

### 2.3. Determination of the Cell Count at the Different Fermentation Time Points

The fermented milk suspension was incubated for eleven hours and colony counts were determined at the beginning (0 h), after 4 h (4 h), and after eleven hours (11 h) of fermentation in three replicates using the layered plating method on MRS agar (Biolab Co. Ltd., Budapest, Hungary). The plating was performed by a 10-times dilution in six steps, depending on the fermentation time points that had the most influence on the colony count. These fermentation time points were determined to be the time zero: 10^1^, 10^2^, 10^3^; fourth hour: 10^2^, 10^3^, 10^4^; eleventh hour: 10^4^, 10^5^, 10^6^, respectively. The plates were incubated for 72 h at 37 °C before counting the colonies.

### 2.4. Analysis of pH

The pH was measured in a controlled environment for 20 h at 37 °C from the fermented milk suspension. The pH change during the milk fermentation was monitored with a Mettler Toledo Seven Multi pH meter every four minutes and resulted in 300 pH values for each fermented milk suspension.

### 2.5. Analysis of the Aroma Composition of the Milk Suspension during the Fermentation Process Using the E-Nose

During the fermentation process, 100 mL of each milk suspension was collected from the fermenting batch for the electronic nose measurements at the beginning, at 4 h, and at 11 h of fermentation and was stored at −18 °C until e-nose measurements. E-nose measurements were performed with a Heracles Neo 300 ultra-fast GC analyzer (Alpha MOS, Toulouse, France). The Heracles e-nose is composed of a rapid, selective, and highly sensitive gas chromatography system that is specifically designed for the analysis of volatile compounds. The system is equipped with an odor concentrator that is called a trap. Its principle of operation is completed when the samples are injected and concentrated in the cold trap, then the trap is heated, and the concentrated odor is injected and divided into two columns—Restek MXT-5: length 10 m; ID 0.18 mm; thickness: 0.40 μm; low-polarity stationary phase; and Restek MXT-1701: length 10 m; ID 0.18 mm; thickness: 0.40 μm; mid-polarity stationary phase (Restek, Co., Bellefonte, PA, USA). Both columns are metal capillary columns, MXT-5 is composed of Crossbond 5% diphenyl/95% dimethyl polysiloxane, while MXT-1701 is composed of Crossbond 14%cyanopropylphenyl/86% dimethyl polysiloxane. The volatile compound is separated by both columns and detected with two flame ionization detectors (FID). The autosampler and the analyzer were operated with the software AlphaSoft ver. 16 (Alpha MOS, Toulouse, France), and the same software was used for data acquisition and basis data transformations. During data acquisition, the retention time of the volatiles was recorded, where retention time is characterized by the elution time of the molecules. Retention times were converted to retention indices. The Kovats retention index compared the retention time of n-alkanes with the retention time of the investigated volatile molecules of a sample under the same conditions [18]. The Kovats index (KI) characterized the volatile compounds on the specific columns and could be assigned to a specific aroma, which was recorded in the AroChemBase v7 [19]. Throughout the manuscript, as an identifier after the KI, the “1A” appears for the column MXT-5 and “2A” for the column MXT-1701. The autosampler’s tray of the e-nose was equipped with a self-developed thermostat which allowed the samples to be stored at 8 °C during the sampling period.

Samples were measured on three different days: on the first day, the samples of the 0th hour; on the second day, the samples of the 4th hour; and on the third day the samples of the 11th hour. The frozen milk suspensions were thawed right before the respective e-nose tests and kept in a water bath at 40 °C for 10 min. Each sample was prepared and injected into the e-nose in three repeats. 

Before the analysis, a method was created with the following parameters of the PAL-RSI Autosampler and the Heracles GC analyzer. Autosampler: 1 g of sample in 20 mL headspace vials with PTFE cap, incubation at 70 °C for 20 min with 500 rpm agitation to generate headspace, 5 mL of headspace injected into the Heracles analyzer, flushing time between injections of 90 s. Analyzer: hydrogen carrier gas, flow of carrier gas at 30 mL/min, trapping temperature at 50 °C, initial oven temperature at 50 °C, endpoint of oven temperature at 250 °C, heating rate at 2 °C/s, acquisition duration of 110 s, acquisition period of 0.01 s, injection speed of 125 μL/s, cleaning phase of 8 min.

### 2.6. Data Analysis

#### 2.6.1. Microbial Assessment

The change of the pH value during the fermentation was approximated with the model described by Torrestiana et al. [20] for each of the tested bacteria strains using the following equation:

pH (t) = (A − D)/(1 + (t/C)B) + D(1)

where: A is the initial culture pH; B relates to the slope of the linear decreasing region from the pH-time curve; C represents the time at which the initial pH decreased to half of its fixed value; D is the final culture pH. The fitted curves were used to determine the pH and fermentation time at the inflection point for each of the strains.

A logarithm with base ten (log 10) of each colony count was calculated and the averaged value of the three parallel samples was recorded for each strain to determine the cell number at the 0th, 4th, and 11th hours of incubation. The initial cell count in the milk suspension was corrected to 10^6^ CFU/mL by dilution based on the cell number determined by the Breed staining method but the layered plating method resulted in slight differences. This was, however, expected due to the relatively high uncertainty of the Breed staining method. Thus, relative colony counts were calculated to reduce the differences that occurred in the initial cell count: the average value of colony counts in 1 mL fermented milk suspension counted at the 4th and 11th hours of fermentation was divided by the average value of the initial colony count for each of the tested strains, separately.

The pH inflection points and relative cell numbers were calculated and illustrated using Microsoft Excel 2013. ANOVA was used to identify any significant differences among the groups of non-probiotic, moderately probiotic, and probiotic strains in the case of relative numbers and inflection points of pH curves. Where ANOVA indicated, a Tukey HSD test (*p* < 0.05) was used for detecting the significant differences between the groups [21]. The ANOVA tests were performed in R-project (ver. 3.6.3) software (R Core Team, Vienna, Austria).

#### 2.6.2. E-Nose Data Construction and Analysis

The results of the e-nose tests obtained during the three days experiment were combined into one file using the AlphaSoft (ver. 16) software (Alpha MOS, Toulouse, France). The chromatograms were transformed into individual variables, in the AlphaSoft (ver. 16) software called sensors, based on the area and respective Kovats indexes of the identified peaks. Classification models were built using linear discriminant analysis (LDA) based on the results of the most selective sensors. Models were built for the discrimination of the strains according to their probiotic activity. This was done separately for the 0th, 4th, and 11th hour. Using the results of the non-probiotic, moderately probiotic, and probiotic strains, LDA models were also built for the differentiation of the different fermentation times (0, 4, and 11 h). The LDA calculations based on the most distinctive sensors were done in the AlphaSoft (ver. 16) software. The models were characterized by the distance and pattern discrimination index (%) between the classified groups.

The results of the e-nose were also analyzed using continuous chromatograms for the two columns separately. LDA models were separately built to classify the groups of the tested strains with regards to their probiotic activity and the three fermentation times. The models were validated with fivefold-cross-validation. The data of the continuous chromatograms were analyzed using R-project (ver. 3.6.3) software.

## 3. Results and Discussion

### 3.1. Growth Characterization of Bacteria Strains in Reconstituted Milk

**Figure 1:**
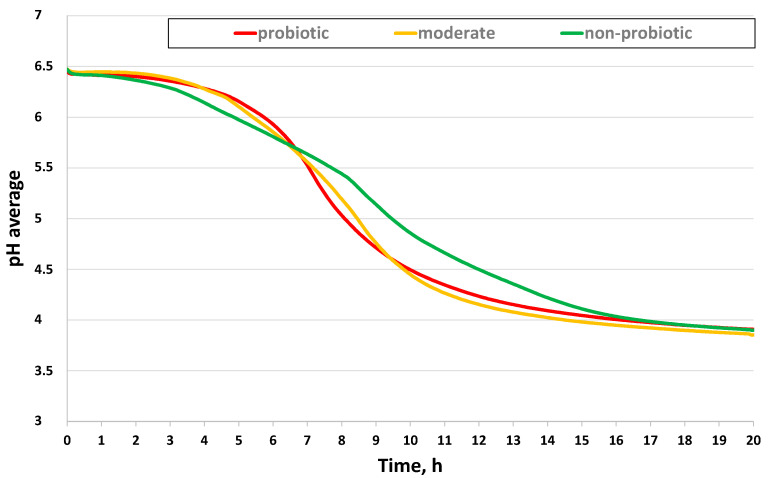
Average pH curves of non-probiotic, moderately probiotic, and probiotic *Lactobacillus* bacteria strains observed during the milk fermentation (*n* = 15).

Results of the average pH change for the probiotic, moderately probiotic, and non-probiotic bacteria groups during the fermentation of milk are shown in Figure 1. A decrease in pH was observed with the increase in the fermentation time. A decrease in pH (from neutral to acidic) was observed from the first hour to the fifteenth. The pH values of the probiotic group decreased at the highest rate especially between the 7th and 9th hours. The general decrease in the pH of all the different bacteria groups can be attributed to the production of lactic acid but the variation in the curves could be due to the different lactic acid-producing activities of the different strains. The pH of all groups, however, became relatively stable after the 15th hour of fermentation when it dropped below pH 4.

Based on the pH curves in Figure 1, the fourth (4th) and eleventh (11th) hours were selected for the comparison of the pH, relative cell count, and aroma composition of the tested bacteria strains. The 4th hour was identified as the lag phase which could be the earliest time to discriminate between the probiotic, moderately probiotic, and non-probiotic groups. On the other hand, the 11th hour coincided with the end of the log phase and was characterized by the highest pH difference between the non-probiotic and the probiotic groups. In addition, this time point was found to be the best for the discrimination of non-probiotic and probiotic candidates in similar studies [9].

**Figure 2:**
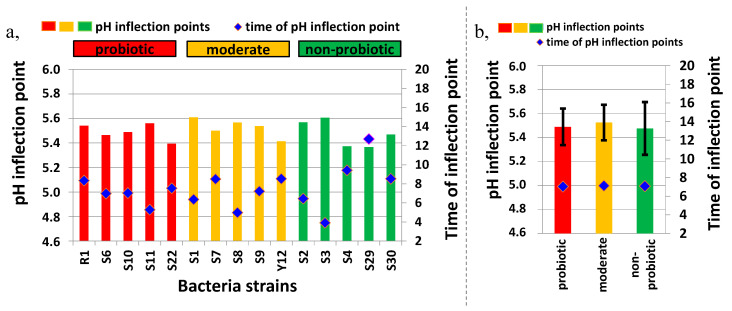
(**a**), Inflection points and time of the inflection points of the pH curves of the tested *Lactobacillus* strains (*n* = 15) and (**b**), an average of the inflection points and time of the inflection points of the pH curves of the non-probiotic, moderately probiotic, and probiotic groups measured during milk fermentation (*n* = 15).

Figure 2a, shows the inflection points of the pH curve and the time of the inflection points for each analyzed bacteria strain, while Figure 2b, presents the average and standard deviation of these values for the non-probiotic, moderately probiotic, and probiotic groups. There were no significant differences found between the inflection points and times of inflection for the analyzed three groups, but the variation in inflection points revealed a lower standard deviation for the probiotic and moderate groups compared to the non-probiotic groups.

**Figure 3:**
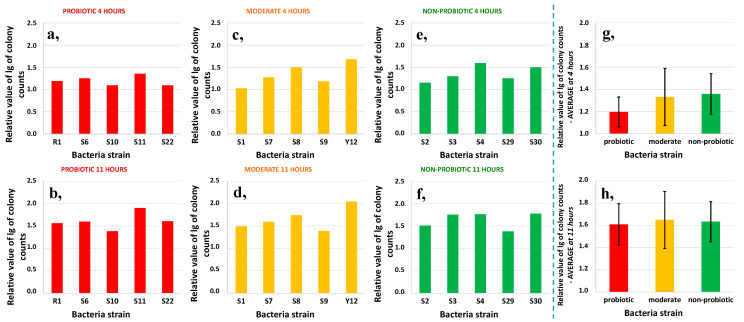
The relative colony counts from the initial 10^6^ CFU/mL number for the tested 15 bacteria strains at 4 h (**a**,**c**,**e**) (*n* = 15) and 11 h (**b**,**d**,**f**) (*n* = 15) and the average values of the probiotic, moderately probiotic, and non-probiotic groups at 4 h (**g**) (*n* = 45) and 11 h (**h**) (*n* = 45) of fermentation in milk.

Colony counts for the 4th and 11th hours of fermentation relative to the initial count of each tested bacteria strain are separately shown in Figure 3. The a, c, and e diagrams show the relative values at 4 h while the b, d, and f show them at 11 hours of fermentation.

Generally, there was only a slight increase in colony counts for all the 15 tested bacteria strains in the first four hours. The increase in colony counts reached 0.5 order of magnitude in only a few cases for moderately probiotic and non-probiotic groups (Figure 3c,e). A further increase in colony counts was observed after 11 h of fermentation for all the strains resulting in 1.5–2 order of magnitude higher colony counts from the 10^6^ initial number. This resulted in an average of 5 × 10^7^ to 10^8^ CFU/mL of the cells after 11 h of fermentation in the milk. Strain Y12 in the moderate group had the highest relative value compared to all the other strains after the 11th hour. This was followed by strain S11 belonging to the probiotic group.

**Figure 4:**
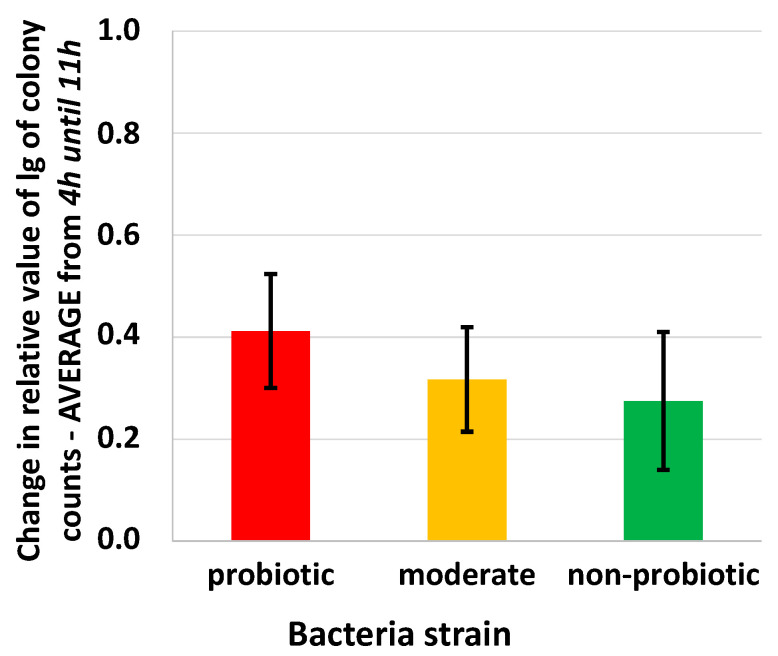
Change in the average relative colony counts from the initial 10^6^ CFU/mL number for the probiotic, moderate, and non-probiotic groups from 4 h to 11 h of fermentation in milk (*n* = 45).

Some differences were observed when the averages of the relative value per colony were calculated (Figure 3g,h) for the different bacteria groups but the differences were not significant. The average relative value of the probiotic group was the smallest at the 4th and 11th hour, but the growth of the moderate group was the most intense at the 11th hour. This agreed with the results of the averaged inflection point for the pH curves. It can be also noted that there was a relatively small increase in colony counts after four hours of fermentation in the probiotic group. This was similar to those of the moderately probiotic and non-probiotic groups after 11 h of fermentation. Figure 4 shows the change in relative values for the three groups from the 4th hour to the 11th. Between the defined time points, the growth of the strains in the probiotic group was the highest and the non-probiotic strains were the slowest. This proves that among all the three different bacteria groups investigated, the probiotic strains provided the highest growth rate when pH started to decrease i.e., in the logarithmic phase of the growth curve.

### 3.2. Discrimination of Probiotic Strains Based on Their Aroma Composition Using E-Nose

**Figure 5:**
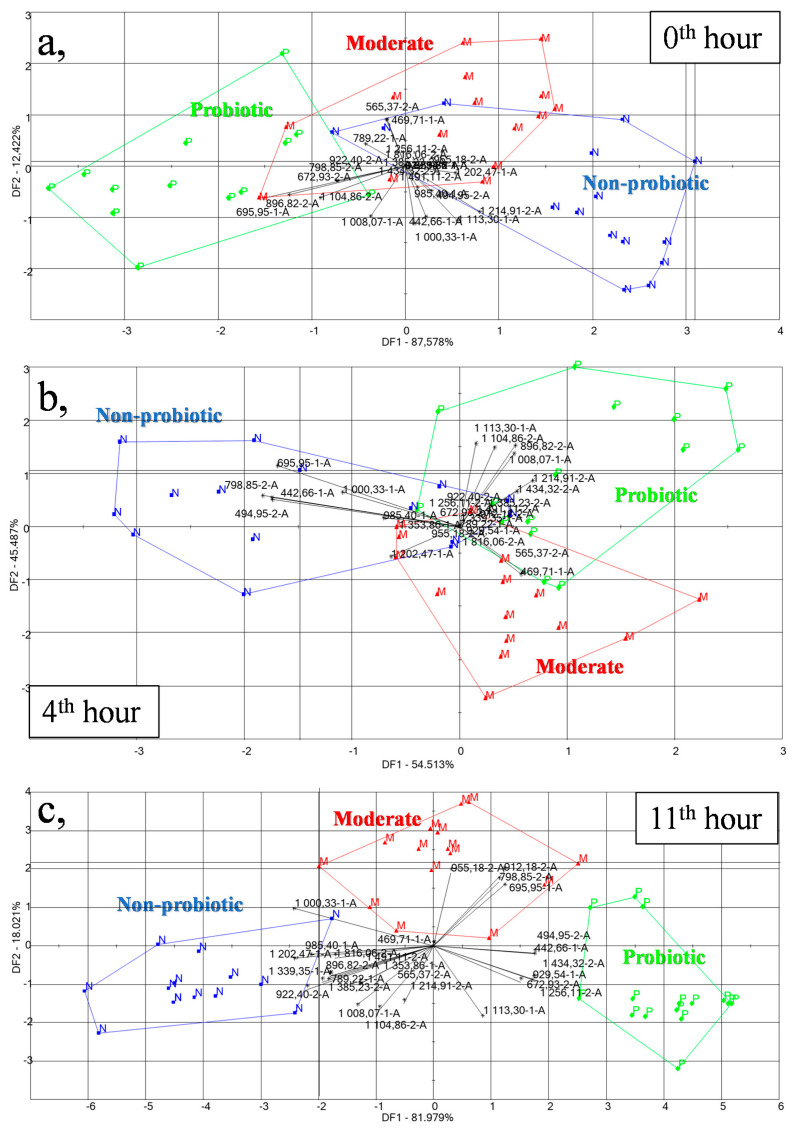
Discrimination of the milk samples inoculated with the 15 bacteria strains based on the probiotic, moderately probiotic, and non-probiotic groups using their aroma composition measured by the e-nose (**a**), at the beginning (0th hour, *n* = 45), (**b**), after four hours (4th hour, *n* = 45), and (**c**), after 11 h (11th hour, *n* = 45) of fermentation.

LDA models built for the discrimination of the non-probiotic, moderately probiotic, and probiotic groups inoculated in milk have been presented in Figure 5. Figure 5a presents the model for the onset of the milk fermentation (0th hour), Figure 5b, after four hours of fermentation, and Figure 5c, after 11 h of fermentation. Improving classification tendency was observed with the longer fermentation time. The average cross-validation score of the samples at the 0th hour was 38% using the 27 selected sensors but increased to 54% in the 11th hour. At the 0th and 4th hour, there was some overlapping between the three groups, but a clear separation was observed at the 11th hour.

Figure 5c shows a complete separation of the three tested groups according to their probiotic activity mainly through the first discriminant factor (DF 1), which describes 81.979% of the total variance between groups. From the left to the right, the tendency according to the increasing probiotic activity was observed. The loading vectors show that sensors 985.4-1A, 1000.33-1A, 922.4-2A, and 1202.47-1A contributed the most to differentiate among the groups. The identified compounds based on the retention indices obtained by the electronic nose are shown in Table 1. Sensor 1000.33-1A can be assigned to the aroma compound of 2-octanol (expressing fatty or oily aroma), decane (sweet aroma), and 2,4-heptadienal (fatty aroma), whereas, 1202.47-1A can be assigned to the compound of pyridine, 2-pentyl (fatty and tallowy aroma). This shows that non-probiotic samples can be characterized more by the fatty aroma. As per literature data, 2-octanol was found during cheese fermentation [22], while 2,4-heptadienal was associated with milk during fermentation [23,24].

In the separation of moderate samples (based on the second discriminant factor), sensor 955.18-2A, 912.18-2A, 798.85-2A, and 695.95-1A had the highest role. Sensor 695.95-1A and 798.85-2A can be assigned to the compound of pentan-2-ol (fruity, green, sweet, pungent) aroma. Pentane-2-ol was also identified during soft cheese fermentation [25]. The loading vectors showed that in the distinction of probiotic samples, sensor 442.66-1A, 929.54-1A, 494.95-2A, 672.93-2A, 1256.11-2A, and 1434.32-2A had the highest role. The latter can be assigned to the epoxy-2-nonenal compound (metallic aroma). The different aromas have also been identified in probiotic yogurts with the GC-MS method [23,26]. Models built based on the continuous data of the chromatograms of the two columns separately showed similar results for the two columns. Classification models built for the discrimination of the non-probiotic, moderately probiotic, and probiotic groups can be seen in Table 2 separately for the three sampled time points. Slightly better classifications were obtained using column 2 (MXT-1701). The average recognition and prediction abilities were 74.44% and 26.66 % for column 1, and 82.78% and 48.99% for column 2 at the 0th hour, respectively. Misclassifications were found in all of the cases, but generally probiotic and non-probiotic bacteria were misclassified as moderately probiotic and vice versa. Probiotic and non-probiotic groups also had 20% and 13.33% interclass misclassification in the case of column 1 and column 2, respectively. At the 4th hour, the recognition and prediction abilities improved compared to the 0th hour for all the bacteria groups. Recognition and prediction were 89.44% and 60% for column 1, and 92.22% and 66.97% for column 2, respectively. At the 11th hour, there was only slight improvement observed in the average recognition and prediction accuracies, however, misclassification between non-probiotic and probiotic groups was further reduced. The average recognition and prediction abilities were 81.67% and 55.55% for column 1, and 81.67% and 59.99% for column 2, respectively.

### 3.3. Discrimination of Inoculated Milk Samples Fermented for Different Times Based on Their Aroma Composition Using the E-Nose

**Figure 6:**
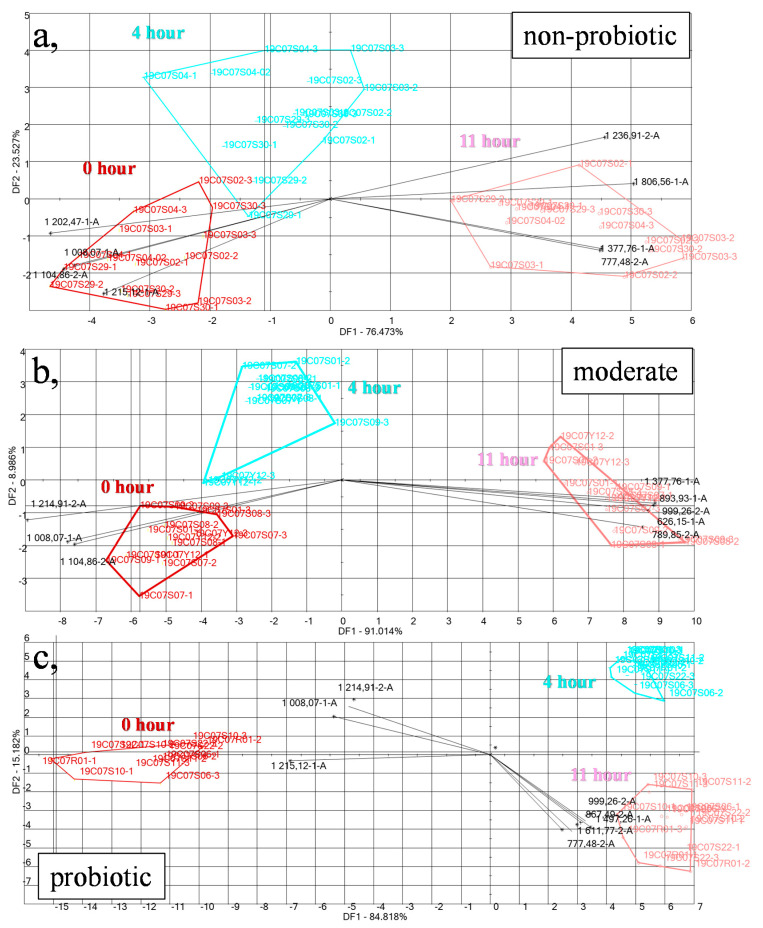
Discrimination of the milk samples inoculated with the 15 bacteria strains based on the incubation time using their aroma composition measured by the e-nose for the group of (**a**), non-probiotic (*n* = 45), (**b**), moderately probiotic (*n* = 45), and (**c**), probiotic (*n* = 45) strains.

LDA models built for the discrimination of the different fermentation times showed a clear separation of the different time periods (0th, 4th, and 11th hour) in the case of all three groups (Figure 6). The model of non-probiotic strains (Figure 6a) showed that sensors 1008.07-1A, 1104.86-2, 1202.47-1A, and 1215.12-1A had the highest role in the differentiation of the samples at the 0th hour from the other two time points. Sensor 1008.07-1A can be assigned to the compound of L-Glutamic acid, N-ethoxycarbonyl-, dimethyl ester; 1104.86-2A to the 2-Propionylpyrrole (popcorn, roast aroma), propionylpyrroline (fishy, roast aroma); 1202.47-1A to the presence of glycine, N-ethoxycarbonyl-, methyl ester, glycine, N-ethoxycarbonyl-, methyl ester, pyridine, 2-pentyl- (fatty, tallowy aroma), beta-Alanine, N-pentafluoropropionyl, propyl ester and acetic acid, (4-(trifluoromethoxy)phenyl)methyl ester, and; 1215.12-1A can be assigned to the presence of L-Alanine, N-pivaloyl, methyl ester, malonic acid, bis(trimethylsilyl) ester and linalyl formate (citrus, coriander, herbasceous aroma). In addition, the loading vectors showed that sensors 777.48-2A, 1236.91-2A, 1377.76-1A, and 1806.56-1A contributed the most to discriminate 11th-hour samples. Sensor 777.48-2A can be assigned to the compound acetic acid (acidic, pungent, sour, vinegar aroma) and n-butanol (cheese, medicinal aroma); 1236.91-2A to the compound 5-ethyldihydro-2(3h)-furanone (coumarin, sweet, tonka broad bean aroma); 1377.76-1A to sentisic acid, O,O’-bis(trifluoroacetyl)-,2,2,3,4,4,4-hexafluorobutyl ester and Propionic acid, 2,3-dimethylphenyl ester, and; 1806.56-1A to the presence of ornithine (N,N,N,O-TMS), L-alanine, 2-methyl-N-(4-methylphenyl)-, 2-methylbutyl ester, glutaric acid, 1,1,1-trifluoroprop-2-yl 2,6-dimethylnon-1-en-3-yn-5-yl ester, and fumaric acid, butyl 2-heptyl ester. Acetic acid has been found in numerous studies as a compound of milk and its products [23,24,27,28,29,30].

The results of the moderately probiotic strains (Figure 6b) showed similar results: sensors 1008.07-1A, 1214.91-2A, and 1104.86-2A contributed to the separation of the 0th-hour group from the other two groups. The separation of samples at the 11th hour was clearer in this case with higher distances compared with the 0th hour.

The loading vectors showed that the sensors 626.15-1A, 893.93-1A 1377.76-1A, 789.85-2A, and 999.26-2A contributed to the separation of the different bacteria groups at the 11th hour. It was observed that the different sensors showed the highest contribution to the 11th hour compared to the 0th hour, showing that the characteristics of the end product may be different if we use moderate strains. In the 0th hour, however, sensor 626.15-1A could be assigned to the presence of compounds of methyl propanoate (fruity, rum, ethereal aroma), 1-propanol, 2-methyl- (alcoholic, bitter, chemical, glue, leek, licorice, solvent, winey aroma), 999.26-2A to the presence of decane (alkane, fruity, fusel, sweet aroma), heptan-2-ol (acrid, fruity, pungent, Roquefort cheese aroma). Heptan-2-ol was assigned previously as a constituent of milk [24,31] and cheese [22,28] and 1-propanol-2-methyl was also associated with milk during fermentation [27]. Sensor 789.85-2A could be associated with the presence of 1,4 dioxane.

Results of the probiotic group (Figure 6c) also proved the phenomena that sensors 1008.07-1A, 1215.12-1A, and 1214.91-2A characterized most of the samples at the 0th hour. This was the same for the non-probiotic and moderately probiotic groups. In the separation of the 11th hour group, sensors 777.48-2A and 999.26-2A contributed the most, these sensors were also assigned in case of the moderately probiotic and non-probiotic groups (Table 1). Nonetheless, new contributing sensors were also found to be present such as 867.49-2A, 1497.26-1A, and 1611.77-2A, resulting in a difference in aroma products. The sensor 1497.26-1A could be assigned to the compounds of DL-malic acid, O-trimethylsilyl-, bis(trimethylsilyl) ester, glutaric acid, 2,2,3,3,4,4,4-heptafluorobutyl isobutyl ester, nicotinic acid, pentyl ester, benzoic acid, 4-(isopropyl) oxy-, methyl ester, benzoic acid, hex-3-yl ester, butyric acid, 2-phenyl-, isobutyl ester. Benzoic acid and nicotinic acids have been also found to be present in milk and milk products and yogurt [31]. Sensor 867.49-2A 3 could be assigned to the presence of 3-hexanone (etheral, fresh, fruity, grape), propyl propanoate (apple, chemical, pineapple), and 1-hexen-3-one (metallic). The metallic aroma was also found to be associated with the probiotic samples at the 11th hour (1434.32-2A).

## 4. Conclusions

Measurements of pH showed a sharp decrease from the 1st hour to the 15th, which could be attributed to the production of lactic acid by the different probiotic bacteria strains. Evaluation of relative values for the different bacteria groups at the 4th and 11th hour showed an increase in the average relative colony counts of the probiotic group in the fermented milk. Bacteria strains S11 and Y13 had the highest relative values after 11 h of fermentation suggesting that those may be the ideal choices for yogurt fortification. The change in relative values was the highest in the probiotic group implying that those were the most sensitive to the conditions of yogurt fermentation.

Electronic nose analysis showed an increasing average recognition and prediction accuracy for the different bacteria groups from the 0th hour to the 4th and 11th. The highest accuracy was observed after 11 h of fermentation, confirming an improved classification tendency with longer fermentation time. Better classifications were obtained using column 2 (MXT-1701) of the electronic nose. The loadings vector of the different classification plots revealed that some of the sensors of the electronic nose could be associated with specific aromas in yogurt. Separate models developed for the acquisition times of the different bacteria groups showed a complete separation of the time points for the probiotic, moderate, and non-probiotic groups. Generally, distances between the time groups were higher between the 0th and 11th hour sample points than between the 0th and 4th or 4th and 11th hours. The electronic nose presents a non-invasive and reliable method for rapid classification and prediction of these parameters.

## Figures and Tables

**Table 1:** Compounds identified by the electronic nose during milk fermentation and their expressed sensory characteristics and comparison with literature.

Identified Compound by E-Nose	Expressed Sensory Characteristic *	Products and References
1-hexen-3-one	metallic	
1-propanol, 2-methyl-	alcoholic, bitter, chemical, glue, leek, licorice, winey	milk [27]
2,4 -heptadienal	fatty aroma	milk [23,24]
2-octanol	fatty, oily aroma	cheese [22]
2-Propionylpyrrole	popcorn, roast aroma	
3-hexanone	ethereal, fresh, fruity, grape	
5-ethyldihydro-2(3h)-furanone	coumarin, sweet, tonka broad bean aroma	
acetic acid	acidic, pungent, sour, vinegar aroma	milk [23,24,27,28,29] cheese [28,30]
benzoic acid, 4-(isopropyl) oxy-, methyl ester, benzoic acid, hex-3-yl ester		[31]
decane	alkane, fruity, fusel, sweet aroma)	
epoxy-2-nonenal	metallic aroma	
heptan-2-ol	acrid, fruity, pungent, roquefort cheese aroma	milk [24,31] cheese [22,28]
linalyl formate (citrus, coriander, herbaceous aroma)	citrus, coriander, herbaceous aroma	
methyl propanoate	fruity, rum, ethereal aroma	
n-butanol	cheese, medicinal aroma	
nicotinic acid, pentyl ester		[31]
pentan-2-ol	fruity, green, sweet, pungent	cheese [25]
propionylpyrroline	fishy, roast aroma	
propyl propanoate	apple, chemical, pineapple	
pyridine, 2-pentyl	fatty, tallowy aroma	

* Characteristics found in the AroChemBase of the E-nose software.

**Table 2:** Confusion table of the LDA models built for the discrimination of *Lactobacillus* strains according to their probiotic activity at the 0th, 4th, and 11th hours using the e-nose.

Column 1 (MXT-5)					Column 2 (MXT-1701)				
**0th hour**					**0th hour**				
Total accuracy		Probiotic	Moderate	Non-Probiotic	Total accuracy		Probiotic	Moderate	Non-Probiotic
Recognition 74.44%	Probiotic	75	15	11.67	Recognition 82.78%	Probiotic	85	15	6.67
	Moderate	16.67	78.33	18.33		Moderate	13.33	81.67	11.67
	Non-Probiotic	8.33	6.67	70		Non-Probiotic	1.67	3.33	81.67
Cross validated 26.66%		Probiotic	Moderate	Non-Probiotic	Cross validated 48.89%		Probiotic	Moderate	Non-Probiotic
	Probiotic	40	40	20		Probiotic	60	46.67	13.33
	Moderate	40	0	40		Moderate	33.33	26.67	26.67
	Non-Probiotic	20	60	40		Non-Probiotic	6.67	26.67	60
**4th hour**					**4th hour**				
Total accuracy		Probiotic	Moderate	Non-Probiotic	Total accuracy		Probiotic	Moderate	Non-Probiotic
Recognition 89.44%	Probiotic	88.33	5	5	Recognition 92.22%	Probiotic	85	0	5
	Moderate	10	95	10		Moderate	5	100	3.33
	Non-Probiotic	1.67	0	85		Non-Probiotic	10	0	91.67
Cross validated 60.00%		Probiotic	Moderate	Non-Probiotic	Cross validated 66.67%		Probiotic	Moderate	Non-Probiotic
	Probiotic	73.33	33.33	13.33		Probiotic	60	20	13.33
	Moderate	13.33	53.33	33.33		Moderate	6.67	73.33	20
	Non-Probiotic	13.33	13.33	53.33		Non-Probiotic	33.33	6.67	66.67
**11th hour**					**11th hour**				
Total accuracy		Probiotic	Moderate	Non-Probiotic	Total accuracy		Probiotic	Moderate	Non-Probiotic
Recognition 81.67%	Probiotic	78.33	13.33	1.67	Recognition 81.67%	Probiotic	78.33	13.33	0
	Moderate	21.67	73.33	5		Moderate	21.67	71.67	5
	Non-Probiotic	0	13.33	93.33		Non-Probiotic	0	15	95
Cross validated 55.55%		Probiotic	Moderate	Non-Probiotic	Cross validated 59.99%		Probiotic	Moderate	Non-Probiotic
	Probiotic	60	46.67	6.67		Probiotic	73.33	46.67	6.67
	Moderate	40	20	6.67		Moderate	26.67	20	6.67
	Non-Probiotic	0	33.33	86.67		Non-Probiotic	0	33.33	86.67
